# Vestibular Evoked Myogenic Potential (VEMP) Triggered by Galvanic Vestibular Stimulation (GVS): A Promising Tool to Assess Spinal Cord Function in Schistosomal Myeloradiculopathy

**DOI:** 10.1371/journal.pntd.0004672

**Published:** 2016-04-29

**Authors:** Júlia Fonseca de Morais Caporali, Denise Utsch Gonçalves, Ludimila Labanca, Leonardo Dornas de Oliveira, Guilherme Vaz de Melo Trindade, Thiago de Almeida Pereira, Pedro Henrique Diniz Cunha, Marina Santos Falci Mourão, José Roberto Lambertucci

**Affiliations:** 1 Graduate Program in Infectious Diseases and Tropical Medicine, School of Medicine, Federal University of Minas Gerais, Belo Horizonte, Minas Gerais, Brazil; 2 Department of Neurophysiology, University Hospital, Federal University of Minas Gerais, Belo Horizonte, Minas Gerais, Brazil; George Washington University, UNITED STATES

## Abstract

**Background:**

Schistosomal myeloradiculopathy (SMR), the most severe and disabling ectopic form of *Schistosoma mansoni* infection, is caused by embolized ova eliciting local inflammation in the spinal cord and nerve roots. The treatment involves the use of praziquantel and long-term corticotherapy. The assessment of therapeutic response relies on neurological examination. Supplementary electrophysiological exams may improve prediction and monitoring of functional outcome. Vestibular evoked myogenic potential (VEMP) triggered by galvanic vestibular stimulation (GVS) is a simple, safe, low-cost and noninvasive electrophysiological technique that has been used to test the vestibulospinal tract in motor myelopathies. This paper reports the results of VEMP with GVS in patients with SMR.

**Methods:**

A cross-sectional comparative study enrolled 22 patients with definite SMR and 22 healthy controls that were submitted to clinical, neurological examination and GVS. Galvanic stimulus was applied in the mastoid bones in a transcranial configuration for testing VEMP, which was recorded by electromyography (EMG) in the gastrocnemii muscles. The VEMP variables of interest were blindly measured by two independent examiners. They were the short-latency (SL) and the medium-latency (ML) components of the biphasic EMG wave.

**Results:**

VEMP showed the components SL (p = 0.001) and ML (p<0.001) delayed in SMR compared to controls. The delay of SL (p = 0.010) and of ML (p = 0.020) was associated with gait dysfunction.

**Conclusion:**

VEMP triggered by GVS identified alterations in patients with SMR and provided additional functional information that justifies its use as a supplementary test in motor myelopathies.

## Author Summary

Schistosomal myeloradiculopathy is a rare and severe form of schistosomiasis caused by *Schistosoma mansoni*, a blood-dwelling worm that lives in the intestinal veins of infected people. The parasite produces eggs that travel in the blood flow and can be trapped in different organs, including the spinal cord. Local inflammation leads to myeloradiculopathy, whose classical symptoms are back pain, numbness and weakness of the legs, erectile dysfunction and urinary retention. Precocious and long-duration therapy with high dose of corticosteroids is necessary to avoid severe sequelae, such as disability to walk. The recommended duration of the treatment varies from three weeks to six months. The physician usually decides when to stop steroids based on physical examination. A neurophysiology exam could improve the evaluation of therapeutic response and the diagnosis of recurrences. Vestibular Evoked Myogenic Potential (VEMP) with Galvanic Vestibular Stimulation (GVS), which is a simple, non-invasive and inexpensive exam, could be used in this context. We assessed the characteristics of VEMP with GVS in patients and found that it was altered in comparison to controls, demonstrating that this exam is a promising tool to add electrophysiological information on the spine to the physical examination.

## Introduction

Schistosomal myeloradiculopathy (SMR) is the most severe and disabling ectopic form of *Schistosoma mansoni* infection [1–3]. Although its prevalence is unknown [1–4], it has been found to be 6% of non-traumatic transverse myelopathies in endemic areas [5, 6]. In SMR, acute transverse myelitis and radiculitis occur as a result of local inflammatory response against the embolized ova in the vessels of the spinal cord, mainly at the lower thoracic and lumbar levels. The anomalous migration of parasites to the central nervous system is explained by a retrograde venous flow into the Batson vertebral epidural venous plexus, which is connected to the portal venous system, were worms are typically located [7–10].

In the acute phase of SMR, the patients present with lumbar and/or lower limbs pain, generally associated with sensitive and motor alterations of lower limbs as well as bladder, intestinal and sexual dysfunctions. If not promptly and adequately diagnosed and treated, the patients remain with serious spinal cord injury sequelae, commonly handicapped, and eventually die because of infectious complications [1, 2, 4, 11]. The diagnosis is based on clinical presentation, evidence of schistosomal infection, magnetic resonance imaging (MRI) and the exclusion of other causes [1–4, 6, 11]. Treatment involves the use of antischistosomal drug (e.g. praziquantel) and corticosteroids [2, 4, 11].

There is no consensus about the recommended doses and duration of steroid therapy [4, 11]. A recommendation of 3–4 weeks using high daily dose of oral steroids (e.g. prednisone, 1.5–2 mg/kg/day) followed by tapering and then complete discontinuation within 3–4 months reduces the risk of severe adverse effects [4]. However, the steroid withdrawal before six months may result in relapse with worse motor sequelae, thus other authors advocate the use of prednisone 1mg/kg/day for 6 months and then start tapering the dose [2, 3, 11]. Hence, the final decision to interrupt corticosteroids may be based on the therapeutic response and not on guideline protocols. The monitoring of the spinal recovery relies mainly on neurological examination, since MRI normalization after treatment may not mirror clinical outcome [2]. Therefore, a supplementary exam is necessary to better guide therapeutic decisions.

Galvanic vestibular stimulation (GVS) is a simple, safe, low-cost and easily reproducible technique used to trigger the vestibular evoked myogenic potential (VEMP) [12–14], which has been used to investigate medullar function in spinal cord injury due to trauma, tumor, ischemia and infection [15–20]. A transcranial GVS is applied on the mastoid bones as a binaural configuration affecting the firing rates of the irregular primary vestibular afferents, exciting on the negative (cathode) and inhibiting on the positive (anode) side of the electrodes [21–23]. The unexpected vestibular stimulus exerts a strong influence on body posture with a protective muscular reflex to maintain postural control:the trunk and limbs sway toward the anode, followed by a counteracting movement [14, 21, 23, 24]. VEMP is the response recorded by electromyography (EMG) in the muscles involved in balance control such as sternocleidomastoid, paraspinal, triceps brachii, tibialis anterior, soleus and gastrocnemius [12–14, 25]. After the GVS appliance, VEMP is captured from muscles of lower limbs through EMG, showing the reflex that crossed the entire neuro-axis, until the lumbar spinal segments. The evoked potential descends through the reticulospinal and the vestibulospinal tracts with similar velocity to the corticospinal tract and integrates motor and sensitivity information [12–14, 19].

VEMP recorded in the soleus or gastrocnemius muscle produces a biphasic EMG wave, with the short-latency (SL) component initiating at approximately 60ms after stimulus onset, followed by the medium-latency (ML) component, which initiates at around 100ms after stimulus onset and is in the opposite polarity of the SL [12–14, 22, 23]. The components of the lower limbs EMG response, although recorded consecutively, do not seem to be related to the same spinal pathway. SL is considered a stable and direct measure of the vestibulospinal reflex, via reticulospinal tract whereas ML is a component of integration, polysynaptic and driven from the semicircular canals to the vestibulospinal tract [12, 16, 23, 25].

The aim of this study was to investigate the spinal cord function of patients with SMR using VEMP with GVS. The results were compared to those of a healthy control group and were crossed with data from neurological examination.

## Methods

### Ethical statement

The Ethics Committee of Federal University of Minas Gerais, Brazil, approved this study (protocol n° 11895813.1.00005149) and it was conducted according to the principles expressed in the Declaration of Helsinki. All subjects gave their informed and written consent.

### Study design and setting

This was a comparative cross-sectional study that enrolled 22 patients with SMR and 22 healthy controls. It was performed between September 2013 and August 2014 at the infectious diseases outpatient clinic of Federal University of Minas Gerais, in Belo Horizonte, Brazil.

### Participants

#### Group of SMR

The group with SMR consisted of 22 patients with definite diagnosis following these criteria: clinical manifestations of myelopathy or myeloradiculopathy; evidence of exposure to *Schistosoma* or positive epidemiology; inflammatory cerebral spinal fluid; MRI with signs of inflammatory myelopathy or myeloradiculopathy; exclusion of other diseases (spinal cord trauma, tumor, vitamin B12 deficiency, antiphospholipid syndrome, diabetic or autoimmune vasculitis, HIV, HTLV, HCV, HSV, HBV, syphilis, tuberculosis, neurocysticercosis, medullary abscess, syringomyelia, herniated lumbar disc, polyradiculoneuritis, demyelinating diseases, radiotherapy) [3, 11].

The 22 included patients were under follow-up at the infectious diseases outpatient clinic of Universidade Federal de Minas Gerais. They had been treated in the acute phase of SMR with praziquantel 50mg/kg and with prednisone 1mg/kg/day for 6 months [3, 11]. To be included in the study, there was no restriction regarding the time of diagnosis of the patients. Exclusion criteria were inability to stand, vertigo complaints and/or neurologic diseases other than SMR [16, 17].

#### Group of healthy controls

The control group consisted of 22 healthy adults, asymptomatic and with no diagnosis of either acute or chronic diseases that could bring physical or psychological limitations. In addition, they had no history of admission to inpatient care facilities for the previous 6 months. Exclusion criteria were vertigo complaints and/or abnormal neurological examination.

### Study protocol and variables of interest

All participants (n = 44) were submitted to anamnesis, clinical and neurological examination. Patients with urinary dysfunction were submitted to urodynamics to confirm neurogenic bladder. Neurogenic bowel was clinically diagnosed if patients presented fecal incontinence and/or fecal constipation with regular need for laxatives, enema or manual maneuvers. Erectile dysfunction was defined according to the Sexual Health Inventory for Men (SHIM) [26]. All participants underwent GVS with EMG recording of VEMP in the gastrocnemius muscle. The VEMP variables studied were the onset (in milliseconds) of short-latency (SL) and of medium latency (ML) waves.

#### GVS procedures

GVS was characterized by a direct monophasic and rectangular 2mA current with pulses of 400ms (model EvP4/ATCPlus, Contronic Ltd., BR). A bipolar current was applied on mastoid bone processes using self-adhesives surface electrodes, with a diameter of 3cm (model CF3200, Valutrode, Axelgaard, Fallbrook, CA, USA). For transmastoid binaural stimulation, both polarities of current were used: cathode right, anode left (CRAL) and cathode left, anode right (CLAR). One trial consisted of 15 CRAL stimuli and then 15 CLAR stimuli, being the inter-stimuli interval randomized between 4 and 5 seconds. Each participant underwent two trials for the right leg and two trials for the left leg [16, 17].

GVS trigged VEMP and the EMG responses were recorded in the gastrocnemius muscle using self-adhesive surface electrodes (model 2223BRQ, 3M, Saint Paul, MN, USA) and one recording channel. The two recording electrodes were vertically placed approximately 5 cm below the popliteal fossa, at a distance of approximately 5 cm between their centers. The reference electrode was placed in the back of the thigh, approximately 5 cm above the recording electrodes. Gastrocnemius was chosen over soleus muscle because of the grater amplitude of the EMG waves [13], facilitating the traces analysis. EMG signals were averaged across 15 stimuli at each polarity (CRAL and CLAR), rectified, filtered between 10Hz and 1,000Hz and digitalized in the sampling frequency of 5,000Hz. Data were collected during successive 500ms periods, starting 100ms before the galvanic stimulus [16, 17].

**Fig 1:**
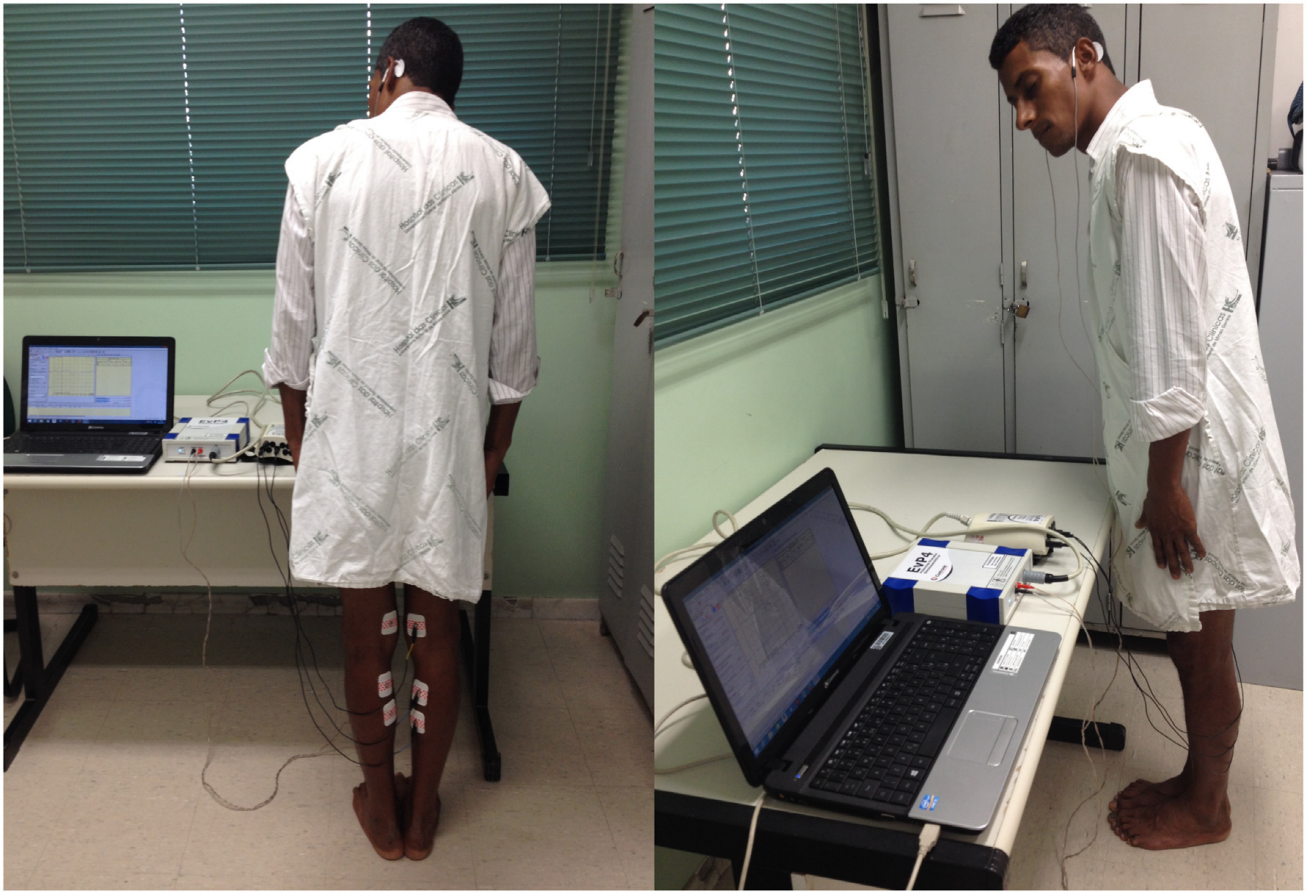
VEMP with galvanic vestibular stimulation being performed in a patient.

During the GVS procedures, subjects remained standing barefoot on a flat surface, with their eyes closed, feet close together and their bodies leaning forward. This position induces the gastrocnemii muscles to keep the contraction and engagement in balance control, which is essential for better evoked responses [12–17]. Subjects were instructed to turn their head approximately 90° to the side contralateral to the examined leg, because the responses are larger and more consistent in the lower limb contralateral to the direction of head rotation [12–17] (Fig 1).

#### SL and ML definition

**Fig 2:**
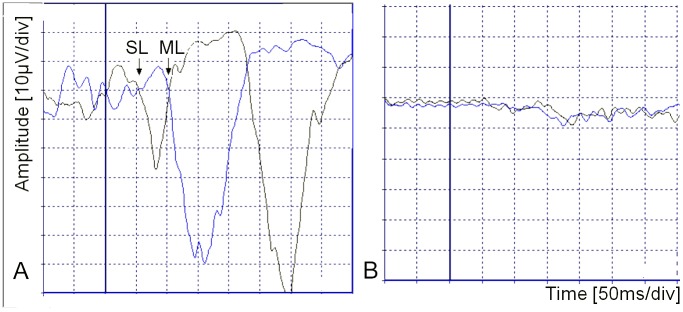
SL and ML electromyographic normal responses to galvanic stimulation in comparison to abnormal responses. (A) Normal responses: superimposed traces of two polarities (cathode right anode left and then cathode left and anode right) reveal inversion of waves and define short-latency (SL) and medium-latency (ML) onsets points. The continuous vertical thick line indicates the galvanic vestibular stimulus onset. (B) Abnormal responses: no identification of SL or ML waves.

An EMG response with onset between 40 and 90ms that inverted with reversal of the stimulus was identified as the SL component. The ML component was subsequent to SL, opposite in polarity and with greater amplitude and duration, occurring at least 90ms after stimulus onset. With the superimposition of the traces with inverted polarity, the point where traces diverged from the EMG baseline marked the onsets of SL and ML, judged visually and measured by the cursor. The first traces-divergence was marked as the onset of SL component. In sequence, traces returned to baseline and diverged again. The second traces-divergence marked the onset of ML component (Fig 2) [12–17, 21–25, 27, 28]. The EMG traces were blindly analyzed by two independent examiners. Besides de latency, abnormal morphology of the wave was a criterion of altered response and the latency was not measured for these cases.

### Sample size

Sample size was calculated *a priori* with the software G*Power 3.1.9.2 to achieve the power of 80% and the significance of 5% based on SL means and standard deviations of patients with HTLV-1 associated myelopathy/tropical spastic paraparesis (HAM/TSP) and healthy control subjects already published [17]. SL mean was chosen over ML mean because of its smaller difference between healthy controls and patients. A minimum of 11 patients and 11 control individuals was necessary. Since no research had been published using GVS in the evaluation of SMR, the sample size was doubled to 22 in each group.

### Statistical methods

EpiData (EpiData Data Entry, Data Management and basic Statistical Analysis System. Odense Denmark, EpiData Association, 2000–2008) was used to build the data bank and SPSS version 15.0 (SPSS, Inc., Chicago, IL, USA) was used for all statistical analyses. Double entered data had asymmetric distribution for all continuous variables, but height (Shapiro-Wilk test). Therefore, non-parametric tests were used for all analyses (Mann-Whitney test for independent samples, Wilcoxson test for dependent samples, Spearman for correlation), with the exception of Student T test for comparison of height between groups. ROC curve was done to analyze the diagnostic performance of SL and ML. The confounders were controlled by linear regression. The level of significance was 5%.

### Potential confounders

The potential confounders age [24] and height [18] did not show any influence on interest variables in the linear regression model.

## Results

### Clinical characteristics of participants

Among the 22 patients with SMR, 17 were male (77%) and the ages varied between 20 and 70 years (median: 42, interquartile range: 28–52). The control group consisted of 22 healthy individuals, 12 male (55%), with ages between 19 to 70 years (median: 30, interquartile range: 26–44). Clinical characteristics of both groups are described and compared in Table 1. The groups were statistically similar regarding age, sex, height and body mass index.

**Table 1:** Characteristics of patients with schistosomal myeloradiculopathy (SMR) compared to healthy controls.

Variable	Patients with SMR (n = 22)	Controls (n = 22)	P value
Age (years)	41.5 [28.0/ 51.5]	30.0 [25.8/ 43.8]	0.162
Men	17 (77)	12 (55)	0.203
Women	5 (23)	10 (46)	0.203
Weight (kg)	70.75 [61.13/ 86.50]	71.00 [59.75/ 84.25]	0.707
Height (m)	1.69 ± 0.10	1.71 ± 0.08	0.432
Body mass index	25.3 [22.3/ 27.7]	24.5 [21.4/ 28.0]	0.534

Data are expressed in median [interquartile range], mean value ±standard deviation or absolute numbers (percentage).

Time of SMR diagnosis ranged from one month to 16 years (median: 61 months, interquartile range: 27–144). Manifestations/sequelae of each patient with SMR are described in S1 Table and their frequency is shown in Table 2. The urodynamics confirmed neurogenic bladder in 13 patients. One presented urodynamics consistent with infra-bladder obstruction. Two refused to do the exam and received the diagnosis of neurogenic bladder based on signs and symptoms of urinary retention with onset at the acute phase of SMR (S1 Table). Three patients were receiving SMR treatment with prednisone 1mg/kg/day (patients 2, 4 and 7; patient 7 was undergoing treatment of SMR recurrence) (S1 Table). The frequency of the affected spinal cord segment is indicated in Table 3.

**Table 2:** Frequency of clinical manifestations of 22 patients with schistosomal myeloradiculopathy.

Clinical manifestations of SMR	N° of patients (%)
**Asymptomatic**	2 (9)
**Lumbar pain**	9 (41)
**Lower limbs pain**	11 (50)
**Gait disturbance**	12 (55)
**Lower limbs paresis**	13 (59)
**Lower limbs hypoesthesia**	14 (64)
**Erectile dysfunction (among 17 men)**	10 (59)
**Lower limbs paresthesia**	15 (68)
**Neurogenic bladder**	15 (68)
**Neurogenic bowel**	16 (73)

**Table 3:** Frequency of affected spinal cord segment of patients with schistosomal myeloradiculopathy.

Affected spinal cord segments*	N° of patients (%)			
	Yes	No	No available data	Total
T1-T6	3 (13.6)	12 (54.5)	7 (31.8)	22 (100.0)
T7-T12	8 (36.4)	7 (31.8)	7 (31.8)	22 (100.0)
Conus	11 (50)	5 (22.7)	6 (27.3)	22 (100.0)
Cauda equine	4 (18.2)	10 (45.5)	8 (36.4)	22 (100.0)

*According to sensitive level and MRI done in the acute phase

### EMG responses to GVS

SL and ML responses were delayed in patients with SMR compared to controls (Table 4). Concerning the morphology of the waves in the SMR group, according to the examiner A, one patient had altered SL and ML and another had altered ML; according to examiner B, three patients had altered SL and four had altered ML. Examiner B did not analyze the EMG of two healthy control individuals. Correlation between two independent examiners measurements was moderate for SL (r = 0.542, p<0.001) and strong for ML (r = 0.834, p<0.001). The area under the ROC curve was 0.814 for SL (p = 0.001) and 0.861 for ML (p<0.001).

**Table 4:** SL and ML in schistosomal myeloradiculopathy patients and controls analyzed by examiners A and B.

VEMP	Examiner	Healthy controls (n = 22)	Patients with SMR (n = 22)	p value
		median and quartiles (milliseconds)		
SL	A	58.73 [55.57/ 60.94]	63.77 [59.68/ 74.18]	0.001
	B	52.35 [49.20/ 56.80]	58.10 [54.30/ 61.80]	0.003
ML	A	108.57 [105.72 / 121.44]	137.57 [122.43/ 152.71]	<0.001
	B	121.55 [101.23/ 133.70]	130.35 [123.60/ 164.00]	0.012

The SL (p = 0.010) and ML (p = 0.024) were more delayed in patients with gait disturbance in the SMR group. No other alteration in the neurological examination was associated with delay in VEMP response.

## Discussion

### Monitoring spinal cord function in SMR using neurophysiology techniques

Neurophysiology techniques are usually used for assessing spinal cord injury, including motor evoked potential with transcranial magnetic stimulation, somatosensory evoked potentials, electroneuromyography, nerve-conduction studies, and motor-evoked potentials as the VEMP [29, 30]. In patients with SMR, VEMP with acoustic stimulation and electroneuromyography have already been investigated [19, 31]. The electroneuromyography was more sensitive to detect schistosomal radiculopathy than the MRI [31]. The limitation of this type of exam in SMR is the lack of direct spinal cord evaluation.

VEMP using acoustic stimulation was shown to be altered in 10 out of 29 (34%) patients with definite SMR [19]. VEMP can be trigger either by acoustic or by galvanic stimulation. The difference is that VEMP with acoustic stimuli generates EMG responses captured in the sternocleidomastoid muscle, innerved by cervical spinal segments, via the medial vestibulospinal tract while the galvanic stimulus generates the evoked response measurable in the lower limbs, innerved by lumbar spine, via the lateral vestibulospinal tract [27]. Taking into consideration that SMR affects more commonly the spine in the lower thoracic, lumbar segments and conus, the galvanic stimulus may be a good supplementary diagnostic tool for follow-up. This is the first study that uses GVS in patients with SMR.

### Assessing anterior and lateral spinal cord function through SL and ML responses

The alteration of VEMP in the lower limbs that was triggered by GVS indicates dysfunction in the reticulospinal and the vestibulospinal tracts, which are located in the anterior and lateral spinal cord [15–17, 28]. CUNHA et al (2013) used GVS to study VEMP in 13 patients with HAM/TSP and found absent waves in around 70% of the patients. When present, the waves were delayed: SL 67±8 and ML 130±3ms in HAM/TSP patients versus SL 55±4 and ML 112±10ms in normal controls (p = 0.001) [17]. In another study testing GVS in 21 patients with spinal cord injury, ILES et al (2004) found 50% of absent VEMP responses. Latency was delayed when the responses were present and the more severe the spinal cord impairment, the longer were the latencies [15]. LIECHTI et al (2008) reported ML with a mean of 130ms in 8 patients with spinal cord injury whereas normal subjects had ML with a mean of 110ms (p<0.050) [16]. These authors did not report SL results because it was often indistinguishable from the baseline [15, 16]. Some level of difficulty in defining SL was also observed in the present study, resulting in only moderate correlation between two independent examiners for SL measurements, whereas correlation was strong for ML. In addition, area under the ROC curve was greater for ML than for SL. Therefore, ML was shown to be the most reliable component of VEMP in lower limbs to define alteration. Others studies confirm the importance of ML, which is considered the wave that represents the motor-sensory integration [12, 15–17, 28].

### Study limitations

The patients with SMR that could not stand were not included, because EMG responses were recorded in the muscles engaged in the maintenance of standing posture. It is possible to record EMG responses from erectors spinae muscles following GVS in sitting patients. In fact, this study was already done to define the level of spinal cord injury [15]. However, in the case of SMR, GVS would not be useful for wheelchair patients. The most important information is about the subclinical functional alterations seen by VEMP while imaging and neurological examination are normal or slightly altered.

### Clinical implications

The diagnosis of acute SMR is based on clinical signs and symptoms, parasitological confirmation and MRI [2–4]. MRI may be used for follow-up, but images frequently normalize after the beginning of the treatment even in patients with incomplete recovery and may not get worse in case of recurrence [2]. In fact, the follow-up of SMR is a challenge since it ultimately relies on neurological examination to guide therapeutic decisions such as the withdrawal of steroids treatment or the reintroduction in case of recurrence. In addition, unresponsive or uncertain cases or even uncooperative patients raise the need for supplementary functional studies. Finally, variables of functional prognostic value could indicate patients who may benefit from longer corticosteroid treatment [2]. In the context of developing countries where SMR and other infectious myelopathies, i.e. HAM/TSP [32] are endemic, a simple, inexpensive and noninvasive neurophysiology technique such as VEMP with GVS can contribute to a better diagnosis and follow-up assistance. Longitudinal studies are going to be done to clarify these hypotheses.

In conclusion, the SL and ML components of VEMP triggered by GVS and recorded in the lower limbs were delayed or absent in patients with SMR, especially in those with gait disturbance. The component ML was more accurate than the SL component. These results showed that VEMP triggered by GVS identified vestibulospinal deficit in patients with SMR. The use of this exam may improve prediction and monitoring of functional outcome during the treatment of SMR by providing additional information on the spinal cord of these patients.

## Supporting Information

S1 Table Neurological characteristics of 22 patients with schistosomal myeloradiculopathy.?: missing data. CE: cauda equina *neurogenic bladder clinically diagnosed (patients refused to do urodynamics). #SHIM: Sexual Health Inventory for Men [1–7: severe erectile dysfunction (ED); 8–11: moderate ED; 12–16: mild to moderate ED; 17–21: mild ED; 22–25: no ED] [26]. NA: not applicable, female patient.
(PDF)

S1 Dataset Dataset of all variables used in the statistical analysis. (SAV)

S1 Checklist STROBE checklist. (PDF)
