# Correction: Vestibular Evoked Myogenic Potential (VEMP) Triggered by Galvanic Vestibular Stimulation (GVS): A Promising Tool to Assess Spinal Cord Function in Schistosomal Myeloradiculopathy

**DOI:** 10.1371/journal.pntd.0004763

**Published:** 2016-05-27

**Authors:** Júlia Fonseca de Morais Caporali, Denise Utsch Gonçalves, Ludimila Labanca, Leonardo Dornas de Oliveira, Guilherme Vaz de Melo Trindade, Thiago de Almeida Pereira, Pedro Henrique Diniz Cunha, Marina Santos Falci Mourão, José Roberto Lambertucci

The following information is missing from the Funding section: DUG and JRL received fundings from Foundation for Research Support of Minas Gerais State (http://www.fapemig.br/) for acquiring the equipment and materials used in this work.
