# Supplementary material for: Vestibular Evoked Myogenic Potential (VEMP) Triggered by Galvanic Vestibular Stimulation (GVS): A Promising Tool to Assess Spinal Cord Function in Schistosomal Myeloradiculopathy
Source: PLoS Negl Trop Dis. 2016 Apr 29;10(4):e0004672. doi: 10.1371/journal.pntd.0004672 (PMC4851389; doi:10.1371/journal.pntd.0004672)
Supplement: S1 Table — CE: cauda equina *neurogenic bladder clinically diagnosed (patients refused to do urodynamics). #SHIM: Sexual Health Inventory for Men [1–7: severe erectile dysfunction (ED); 8–11: moderate ED; 12–16: mild to moderate ED; 17–21: mild ED; 22–25: no ED] [26]. NA: not applicable, female patient. (PDF) [file pntd.0004672.s001.pdf]

Patient	Affected spinal cord segments	Asymptomatic	Neurogenic bladder	Neurogenic bowel	Erectile dysfunction (SHIM)#	Lumbar pain	Lower limbs pain	Lower limbs hypoesthesia	Lower limbs paresis	Lower limbs paresthesia	Gait dysfunction
1	?	-	+	+	NA	+	+	-	-	-	-
2	?	-	+	+	+ (21)	+	+	+	+	+	-
3	T6-S5 +CE	-	+	-	-	-	-	-	-	-	-
4	T8-T10	-	+	+	+ (4)	-	-	+	+	+	+
5	T10-S5	-	-	-	-	-	-	+	-	+	-
6	L1-S5+CE	-	+	+	NA	-	+	+	-	+	-
7	T9-S5 +CE	-	+	+	+ (1)	+	+	+	+	+	+
8	L1-S5	-	+*	+	+ (4)	+	-	+	+	-	+
9	T6-T9	-	+*	+	+ (7)	+	-	-	+	+	+
10	T10, L1-S5	+	-	-	NA	-	-	-	-	-	-
11	?	-	+	+	+ (9)	-	+	+	+	-	+
12	L1-S5	-	+	+	+ (1)	+	+	+	+	+	+
13	L1	-	+	+	+ (1)	+	+	-	+	+	+
14	?	-	+	+	NA	-	-	+	+	+	+
15	L1-S5	-	+	+	+ (7)	-	+	+	+	+	+
16	?	-	-	-	+ (7)	+	+	+	+	+	+
17	?	-	-	-	-	-	-	+	-	-	-
18	?	-	-	+	-	-	-	-	-	-	-
19	L1-S5 + CE	-	+	+	NA	-	+	+	+	+	+
20	T4-T12	-	+	+	-	+	+	+	+	+	+
21	T8-T11	-	-	+	-	-	-	-	-	+	-
22	T6-S5	+	-	-	-	-	-	-	-	-	-
